# How to use relevant data for maximal benefit with minimal risk: digital health data governance to protect vulnerable populations in low-income and middle-income countries

**DOI:** 10.1136/bmjgh-2019-001395

**Published:** 2019-04-11

**Authors:** Nicki Tiffin, Asha George, Amnesty Elizabeth LeFevre

**Affiliations:** 1 Wellcome Centre for Infectious Disease Research in Africa, University of Cape Town, Cape Town, South Africa; 2 Computational Biology Division, University of Cape Town, Cape Town, South Africa; 3 Centre for Infectious Disease Epidemiology and Research, Public Health and Family Medicine, University of Cape Town, Cape Town, South Africa; 4 School of Public Health, University of the Western Cape Faculty of Community and Health Sciences, Cape Town, South Africa; 5 Division of Epidemiology and Biostatistics, Public Health and Family Medicine, University of Cape Town, Rondebosch, South Africa; 6 Department of International Health, JohnsHopkins Bloomberg School of Public Health, Baltimore, Maryland, USA

**Keywords:** digital health, mHealth, consent, governance, ethics, LMICs, low and middle income countries

## Abstract

Globally, the volume of private and personal digital data has massively increased, accompanied by rapid expansion in the generation and use of digital health data. These technological advances promise increased opportunity for data-driven and evidence-based health programme design, management and assessment; but also increased risk to individuals of data misuse or data breach of their sensitive personal data, especially given how easily digital data can be accessed, copied and transferred on electronic platforms if the appropriate controls are not implemented. This is particularly pertinent in low-income and middle-income countries (LMICs), where vulnerable populations are more likely to be at a disadvantage in negotiating digital privacy and confidentiality given the intersectional nature of the digital divide. The potential benefits of strengthening health systems and improving health outcomes through the digital health environment thus come with a concomitant need to implement strong data governance structures and ensure the ethical use and reuse of individuals’ data collected through digital health programmes. We present a framework for data governance to reduce the risks of health data breach or misuse in digital health programmes in LMICS. We define and describe four key domains for data governance and appropriate data stewardship, covering ethical oversight and informed consent processes, data protection through data access controls, sustainability of ethical data use and application of relevant legislation. We discuss key components of each domain with a focus on their relevance to vulnerable populations in LMICs and examples of data governance issues arising within the LMIC context.

**Summary box**

- Digital health data provide both opportunities for benefit and risks for vulnerable populations. We propose a data governance framework that can both reduce the risk of digital health data misuse while promoting increased access for potential benefit.
- Our primary aim is to provide a framework that will assist stakeholders to understand the key elements required for good data governance within digital health systems.
- We present four key domains within this framework, namely (1) ethical oversight and informed consent processes, (2) data protection through data access controls, (3) sustainability of ethical data use and (4) application of the relevant legislation.

## Introduction

Individuals commit a growing proportion of their personal and private data to digital devices during routine use; and simultaneously, technological advances for saving, storing, duplicating and transferring digital data mean that replicating and sharing datasets has become much easier to facilitate. Health systems can leverage these data and bring evidence-based depth to intervention design, programme management and performance assessment. This has led to a rapid expansion of technology use in the health sector to both generate and share large, granular and informative data. These advances in digital approaches to data open avenues for rethinking how we handle data across health systems levels to advance the health of vulnerable populations in low-income and middle-income countries (LMICs).

Near ubiquitous access to mobile phones has raised the profile of mobile phones as tools for improving patient-provider communication, access to health services and information and data collection (reviewed in Ref. 1). Rapid implementation of mobile and digital tools in the health sector, however, has triggered concerns. The digital health ecosystem is particularly vulnerable to data misuse because it combines extremely sensitive health data with digital platforms that are well suited to replication and dissemination of datasets. With an online personal computer or mobile device it is possible to copy and disseminate huge datasets almost instantaneously, which increases the risks of inappropriate data sharing, and makes it harder to contain or reverse data breaches: the stakes are much higher for digital datasets because once shared, it is almost impossible to track down or delete copies of those data. This is further exacerbated by the complexity of the data flow involving multiple channels with a range of stakeholders and points of exposure—from individuals to data consumers, via data collectors; through mobile devices, interoperability layers and intermediate databases; and to databases where the data are permanently held.^2^ Finally, the potential for unconsented commodification of collected data, whereby individuals cannot control or access how their data are being shared, reused or commercialised, poses a significant risk. Collectively these risks are particularly heightened in low-resource settings marked by deep intersectional inequalities, and where governments are lagging behind in implementing data protection policies and regulatory oversight to ensure protection of personal information.

Given emerging opportunities and risks in digital data use, here we propose a data governance framework to reduce risks of data misuse while promoting increased access for potential benefit, in the context of expanding scope, depth and coverage of data-driven digital health interventions. We present four key domains in which data governance structures can be articulated and implemented to ensure appropriate data stewardship: (1) ethical oversight and informed consent processes, (2) data protection through data access controls, (4) sustainability of ethical data use and (4) application of relevant legislation.

Our primary aim is to provide an overview that will assist stakeholders to understand the key elements required for good data governance within digital health systems (summarised in box 1), so as to ensure maximal benefit while meeting universal ethical standards. This framework is derived from our own experiences working with digital health data in South Africa and India, and is intended to provide a practical framework to assist others similarly developing their own data governance structures. We also highlight key elements from legislation on data protection that are relevant to health programmes. We illustrate our framework with examples drawn from LMICs, and our experience working in programmes in South Africa and India, but we believe that the principles are universal.

Box 1 **A checklist for implementing digital data governance principles**

**Ethics and informed consent**

- Vulnerable populations are identified and appropriate resources assigned for protection of their data.
- Tiered consent process is clearly delineated and each level of consent is stored.
- Patient information describes in detail intended data use, storage and future destruction.
- Option to withdraw from study with data deletion is clearly outlined for participants.

**Data access**

Procedural oversight

- Put in place clear procedures for processing data access requests which include oversight by key stakeholders.
- Define protocols to guard against data commodification.
- Articulate important metrics for assessing access requests, which may include:
  - Geographic locations of data requestor and requested data.
  - Fair representation of all stakeholders with sensitivity to postcolonial inequities and appropriation.
  - Providing minimum data to service requests without unnecessary exposure of sensitive data.
  - Maximising permissible benefit from appropriate data use.
- Avoid person-centric gatekeeping around data and establish committees, standard procedures and guidelines for data use together with government stakeholders.

Structural controls

- Install appropriate remote-delete software on devices in case of loss or theft.
- Restrict app installations and personal use on devices used to collect participant data.
- Separately store and transport identifying and sensitive/clinical data.
- Store data in secure, firewall-controlled and access-controlled locations.
- Where possible work within secure digital environments used by local health departments.

**Sustainability**

- Build an interoperable data structure so that data can be easily shared where appropriate.
- Provide up-to-date documentation, consent information and codebooks for all datasets.
- Establish a data backup plan for frequent back up to secure locations.
- Implement a long-term data storage and management plan that is not dependent on particular individuals or organisations.

**Legal Framework**

- Familiarity with relevant sections of all local/regional legislation pertaining to Healthcare, Protection of Privacy, Access to Personal Information Acts.
- Identify the entity responsible for the data and key stakeholders, in collaboration with government structures.
- Facilitate review by local regulators where necessary.
- Comply with restrictions on moving data across borders, including identifying related issues with Cloud storage.

## Key tenets for data governance

**Figure 1:**
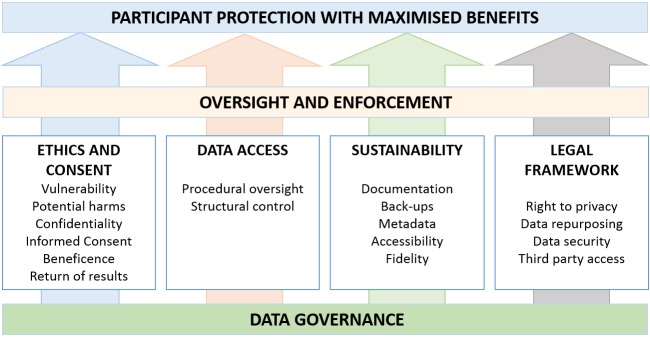
Pillars of digital data governance to ensure protection of individuals.

Figure 1 presents the four main pillars of digital data governance that we believe are critical to ensure health benefit and participant protection for vulnerable populations in LMICs. We provide further detail for key elements that fall within each of these domains.

### Ethics and informed consent

Below, we discuss key elements of ethics considerations for participant protection, with a focus on how these elements may impact the use of digital health data from vulnerable populations. We recognise the importance of community engagement and public involvement in informing these ethics considerations prior to embarking on the collection of digital health data.

#### Vulnerable populations

As part of participant protection, vulnerable populations must be identified and special care taken when requesting data from these participants. In LMICs, the likelihood of participants having some level of vulnerability is high, and this is exacerbated in areas of conflict and humanitarian crisis.^3^ While digital technologies can increase access to key populations, where participation takes place through digital platforms without a human interface there is an added layer of difficulty to ensuring appropriate protection and ethical engagement with vulnerable individuals. In contexts where a human interface is available, the nature of that interaction coupled with the characteristics of the individuals involved—including their education, language, the presence of power differentials and other factors—may drive participation and engagement, including perceptions on whether it is compulsory. In the context of South Africa’s national mobile maternal messaging programme ‘MomConnect’, while current registration processes via healthcare providers during antenatal care clinics provide an opportunity to obtain informed consent and for providers to answer participant questions, it too raises important questions about whether participants can decline participation and what (if any) the consequences might be on the quality of care received and/or future health systems contacts.

#### Potential harms

While potential harms in the field, for example in a clinical trial, might be tangible and physical, potential harms through the use of personal and health data can be harder to predict. Health data breaches can lead to stigmatisation of individuals or a demographic group,^4^ and unconsented secondary use of personal data may also lead to privacy infractions through unsolicited contact or content—which may have unintended consequences. For example, phone numbers are collected through public sector Maternal Child Tracking System and Reproductive Child Health registers in India and may be used for the passive enrolment of women into mobile health information campaigns. Delivery of these messages, including those pertaining to family planning, on shared phones may result in family misunderstandings and conflict.^5^

Understanding the representativeness of the population for whom digital data are being collected is vital in ensuring its responsible use. Harm can unintentionally occur when data drawn through digital means are assumed to be generalisable when they are not. Such an assumption may further increase inequities in the representation of needs and distribution of resources by building on existing differentials in access to mobile phones and other digital tools, as well as variations in digital literacy among those with access to a mobile phone and other digital tools.

Importantly, it is not always possible to predict future risks to individuals or communities within the limits of current knowledge. For example, the concept of one’s own data privacy choices inherently implicating others is gaining traction through recent high profile use of online genomic data resources to identify relatedness.^6^ The increasing ubiquity of mobile phones and tablets in the hands of frontline healthcare providers increases risk of health data exposure through concurrent personal and professional use of devices: household phone sharing, theft of the device, or exposure to malware and viruses from downloaded applications could lead to unintended health data disclosure by the healthcare worker.^7^

#### Confidentiality

A key requirement for ethical research is to ensure that data from participants remain confidential, and the changing data landscape needs to be met with enhanced approaches to confidentiality. Paper records might be locked in filing cabinets with physical access restrictions, but for electronic data the restrictions must be technically appropriate to ensure that the confidentiality entrusted by participants is upheld. Suitable approaches include system administrative and firewall restrictions on who can access electronic data resources and encryption of drives containing sensitive data. Data de-identification or anonymisation has been a standard approach to ensure participant confidentiality and reduce risk of disclosures, but re-identification risks increases with as data becomes ever more granular.^4^ Anonymisation is harder to ensure—a problem especially well illustrated by efforts to de-identify genomic data which are intrinsically and ultimately defining of an individual. Even aggregated data reporting using combined measurements for groups of individuals can result in stigmatisation, ‘othering’ or negative stereotyping of demographic groups, and geocoded data can increase this risk through geographical localisation, for example, ‘hotspot mapping’ for prevalence of infectious pathogens such as HIV or tuberculosis. Reviews of community acceptance of data collection in LMICs suggest that participants may also doubt the confidentiality of their data collected electronically (reviewed in Ref. 8); and the paucity of reports on breaches of confidentiality in LMICs suggests that oversight, breach detection and reporting are lacking.^9 10^

#### Informed consent processes

Key items related to digital data that should be covered in the information section for participants undergoing informed consent include:

1. The intended data use: by whom and for how long. Primary, secondary and general data use should be clearly indicated, and separate (tiered) consent obtained for these. This should include details of any intended monetised secondary use of or third party access to the data.
2. If, and how the data will be anonymised for further analysis.
3. How the data will be protected, and when the data will be destroyed.
4. Risks and benefits to the participant, including any remuneration.
5. Contact information for further questions or concerns and/or requests to withdraw from the study, and information about how data will be deleted at the end of the study.

As programmes and legislation expand and evolve over time, changes to the content and processes for obtaining informed consent may be required; and adjustments made. Box 2 illustrates the informed consent process for the MomConnect programme in South Africa, highlighting how such consent processes may be affected by implementation of the Protection of Personal Information (POPI) Act in South Africa,^11^ as well as unanticipated ways in which the process of obtaining consent changed over time to accommodate programme integration with routine health services delivery.^12^

Box 2 **Implications for MomConnect of legislative change and the protection of personal information (POPI) Act in South Africa**

**Consent procedures**

The current MomConnect programme consists of three components: (1) pregnancy registration, (2) delivery of health information messages and (3) helpdesk. Registration to MomConnect occurs during antenatal care in the public sector, where oral consent should be taken by a healthcare provider.

- Registration fields include a USSD consent message: ‘We need to collect, store, and use her. [pregnant woman’s] info. She may get messages on public holidays and weekends. Does she consent? 1. Yes; 2. No’.
- Additional data elements collected as part of registration include women’s phone number, expected date of delivery, language preference, facility code and at least one of the following: date of birth, identification type (telephone, national id or passport number) and identification issuing authority (country).

**Unanticipated adaptations**

- In 2016, over half of registrations occurred on a device other than the women’s personal mobile phone, including the healthcare provider or a facility-based data entry clerk.^12^
- High patient volumes, clinical demands on providers, coupled with the lengthy time required to register women to MomConnect over USSD has meant that registration often occurs in ‘batch’ on a device other than the women’s personal mobile phone. In practice, this means many of the registration details are captured on paper and later input by a designated data entry clerk or provider in the clinic.
- This unfortunately means that for half of all women registered, their consent cannot be confirmed because these data were not collected on their personal mobile device.
- Qualitative interviews suggest that there are often inaccuracies in the data inputted during registration, including user preferences for language (A E LeFevre, personal communication).

**How might consent procedures be enhanced?**

- Women are currently consenting to have the data collected during registration for undefined purposes. Consent language should be modified to more comprehensively capture the intended uses for the data and sent to the participant on their own phone.
  - Suggested language: ‘We need to collect, store and use your information for sending you text messages containing health information about your pregnancy. You may get messages on public holidays and weekends. Do you agree for us to use your information this way? 1. Yes; 2. No’.
  - To use data for purposes other than the delivery of health information content, further revisions to consent language would need to be made to capture anticipated uses.
- For individuals registered on a device other than their own, an SMS text message is sent separately to them on their own mobile phone to obtain consent. Only those responding in the affirmative (and thus ‘opting-in’ after registration details are collected) should be registered to the programme.
- A system must be put into place for registered users to access data and update where needed. The procedures for accessing these data must be communicated to users and readily available.
- Use of data for research purposes must be governed by accepted standards for human participants research.

**What are some further requirements for POPI compliance?**

- The responsible party for the data should be identified to participants, with contact details in the event of any participant concerns.
- Any additional data use beyond provision of maternal text messages must be explicitly communicated and consented (this would apply for research) at the time of registration. Understanding that not all potential uses of data can be anticipated from the outset of a programme, consent language may need further refinement as additional data use scenarios come arise.
- Any intended third party data access must be communicated and explicitly consented to by the participant at the time of registration.
- Explicit consent must be requested for any future unsolicited contact.
- A clear process must be communicated to opt out and have records deleted at any time, with reassurance that normal standard of care will be received and there will be no negative consequences of declining or opting out.

#### Beneficence

Assessing beneficence of a programme or study generating digital data requires an understanding of how the participant (or other people) may benefit from those data. To be considered ethical, the output must be demonstrably truly beneficial either to participating individuals, or for the common good. Given that a study is sufficiently well-designed, the extent of beneficence must be offset against—and substantially outweigh—the concomitant potential for harm to participants through data breach or misuse. Furthermore, the benefits must be equitably distributed. Participation in international health systems research can be tainted in inequality and surface in postcolonial research dynamics.^13–15^ Other concerns include benefit of data use for well-resourced populations at the cost of those who are under-resourced;^16–19^ unequal distribution of funding for data-generating research^20 21^ and silo’d aid programmes that operate in parallel with local health systems or fail to return insights or benefits from participant data that they collect.^21 22^

#### Return of results and secondary findings

Balancing beneficence and risks also encompass returning study results or secondary findings from data reuse to all stakeholders including field workers, health departments, health client populations and study participants. Such feedback loops are often overlooked by researchers and programme implementing partners alike—especially where researchers do not have direct interactions with participants because of digital implementation. There appear to be few frameworks for data return. Furthermore the logistical challenges of recontacting study participants to share findings can be particularly challenging in LMICs. Frameworks for data return to individuals should be established at programme outset, adequate resources (time and costs) allotted and specific consents should be requested from participants: not everyone wishes to receive personal data findings, and secondary findings can cause distress and discomfit, especially when a definitive or actionable result cannot be provided.^23 24^ Decisions must be made about how secondary findings will be classified as appropriate for return to participants, accordingly.

### Data access controls

Once data are generated, the responsible party which commissioned the collection of the data must ensure that they are managed in an appropriate and legal way. Access to digital data can be managed at two levels, explained below. Procedural controls ensure that documented processes are in place to ensure data protection and appropriate reuse; and structural controls use information technology and infrastructural and technical protections to guard against data breach.

#### Procedural oversight for data access

Once a dataset has been generated, it is advisable to establish a clearly articulated, unbiased process to apply for data access. This may involve submitting a request to a formalised Data Access Committee or similar board, who should also keep a full recorded history of data access granted and datasets disseminated. Standard operating procedures for how to access data; and clear, transparent documentation of the process should be openly available. Some key issues that may be addressed during the review of a data access request might include:

##### The geographic location of the requestor of the dataset and the origin of the data

Governments have differing legislation over how data may be transferred across borders, and this must be clearly articulated in consideration of data access requests; for example in South Africa, transfer of identified/identifiable health data of South Africans outside the country can only be undertaken with specific permission by the POPI regulator.^11^ This includes scenarios where data may be requested through cloud storage, where the physical location of the cloud server may also need consideration.

##### Whether the data request submitted is equitable

Data access requests should include a fair representation of all partners and be sensitive to postcolonial inequities and appropriation.^13 14^ Some data access committees may require some form of contribution to the data-generating site or partnerships that lead to capacity or skills development to discourage inequitable or predatory data acquisition. For example, where a health department in an LMIC has the skills and resources to undertake effective analysis of their own data, a request for the data from an academic researcher who has no existing relationship to the health department, country or dataset may be reconsidered.

##### Whether the data use requested allows maximal permissible benefit to be obtained from the dataset while minimising participant risk

The Data Access Committee should also consider the ethical use of the generated data,^25^ balanced with risks and beneficence for participants. For example, requested access to a large and complex dataset—exposing participants to some risk of data breach—to answer a simple research question of limited importance or applicability might be discouraged.

#### Structural implementations for data access control

Structural and technological protocols may also be employed to protect data. These include software-based solutions such as firewalls, encryptions, passwords and systems administration to control access to databases and datasets stored on servers or computers. Database design can also increase protection of participants, for example by separating biometric and identifying data in a different database to clinically informative data. In this way, sensitive data cannot be easily linked to identifying data and a separate linkage key is needed to join physically separated data. Tiered or partial data access can be provided as appropriate, and a ‘minimum data’ access policy can prescribe that individuals only have access to the data that they absolutely require to see.

Additional structural governance items include implementation of secure back-up and disaster recovery plans, with appropriate security for stored copies of data accordingly. Where data must be transferred, well-documented data transfer protocols should exist, describing encryption, password protection, separation of sensitive from identifying data for transfer and the use of secure platforms for data transfer rather than email, flash drives or other insecure transfer media. A recent literature review has shown, for example, the proliferation in LMICs of use of the WhatsApp chat application for sharing patient information between clinicians—with scant regard for security, consent or protection of confidentiality.^26^ This may be fuelled by a lack of accessible, convenient and secure internal platforms for efficient and appropriate sharing of patient data.

### Sustainability

Effective data governance requires a clear, documented plan for sustainability, to ensure maximised benefit and minimal risks from health systems data into the future. This arises from an ethical imperative to ensure that the data can be reused where appropriate and where ethics are in place, ensuring maximised return for funding—especially when funded by public and taxpayers’ money and also out of respect to participants for the time, effort and risk they have endured in order to provide the data. A formalised sustainability plan can ensure that outcomes are maximised and achievements can be far-reaching and sustainable without compromising other processes.

As the use of digital tools has grown throughout the last decade, in LMICs they have proliferated in vertical and silo’d digital health programmes, competing for finite resources, often duplicating prior efforts, and in some cases diverting precious resources from core health services delivery. In these cases, parallel healthcare streams are not integrated with national or governmental health resources, and the digital health data they collect are not harnessed for strengthening core health systems and are at risk of inappropriate reuse. In the digital health ecosystem, the expansion of disease or condition specific small scale apps and digital health solutions has led to a fragmentation of tools designed with limited interoperability or extensibility. Further, in many contexts, design and implementation are led by technology companies with limited clinical or public health personnel. The lack of planning for sustainability, integration into existing health information systems and interoperability has led to ‘pilotitis’—the proliferation of short lived standalone mobile and other digital health tools. In response, some governments (eg, Uganda^27^) have called for moratoriums on new app deployment within the health system. In under-resourced environments in LMICs, health systems monitoring or evaluation programmes should rather ensure integration with core health services, scalability and a long-term trajectory for the programme.^28–31^

Sustainability planning requires having systems in place to ensure that data stewardship is entrenched and is not personality-driven but rather systemic and person-agnostic. In the technology sector, turn-over of staff can be high which can result in loss of institutional and programme knowledge unless a sustainability plan is in place. It requires the data to be consistently, clearly and extensively documented, and properly stored and backed up. Furthermore, datasets should adhere to FAIR principles for datasets by appropriate recording of dataset metadata:^32^ they should be Findable, Accessible, Interoperable and Reusable (where ethics approvals and informed consent are in place for the data to be used further). Adhering to FAIR principles will ensure that data can be retrieved, queried and reused into the future.^32 33^ Planning for sustainability requires upfront budgeting to ensure that data remain accessible and useable for authorised end-users, and sufficient resources should be allocated accordingly.

### Legal frameworks

Legislation on POPI has been drafted in many countries and is continually being reviewed and refined as the proclivity for big data increases globally. It is, however, challenging for legislators to predict and anticipate new types of potential data misuse; and evolving risks cannot always be anticipated as new data types evolve. Concerns with recent high profile personal data breaches have prompted revision of legislation and oversight in many countries. For example, in India concern about substantial data breach of the Aadhaar biometric identification system has resulted in revisions of the Healthcare Security Act.^34–37^

Another challenge alongside such iterative refinement is that implementing oversight and enforcement are not yet commonplace. As a result, legislation tends to be reactive to challenges rather than pre-emptive, so data generators and consumers need to take the initiative in ensuring good data governance within the framework of successful, data-driven health systems development and implementation.

In South Africa, the POPI Act is currently being implemented, with specific legislation detailed for health data, which are considered ‘special’ data requiring particularly stringent regulations for use and reuse.^11^ Furthermore, the Health Act of South Africa^38^ defines the confidentiality that must be upheld between clinician and client, ensuring protection of the client’s sensitive data; and the Promotion of Access to Information Act (PAIA)^39^ enshrines the right for individuals to be able to access and review data held about them: every individual should be able to receive a full account of how their data have been used, on request. Ensuring compliance with PAIA requires suitable logging and storage of data usage, so that this can be provided on demand. The European Union has recently implemented a progressive legislative framework for protection of personal information,^40^ which may also inform new legislation in other countries. Online supplementary table S1 compares examples of content areas addressed by legislation in the European Union and South Africa, and online supplementary table S2 compares and explains some key terminology used by both. A more detailed comparison of some key sections of the legislation is provided in online supplementary table S2.

10.1136/bmjgh-2019-001395.supp1 Supplementary data

## Oversight and enforcement

The framework that we have presented here proposes oversight of data access through data access committees, oversight of ethical compliance by ethics committees and compliance with legislation through legal infrastructure overseen by legislative infrastructure—for example, data protection officers mandated by GPDR in the European Union.

To ensure the implementation of these components of good governance, structures for enforcement are also needed. In the context of national health initiatives or public health programmes, a Department of Health committee may exist which provides broad support to implementation, ensuring immediate adherence to legislative standards and providing guidance on practical steps for implementation—for example, in the context of the MomConnect Program in South Africa, a Department of Health Task Force chaired by the a full-time senior advisor serves in this capacity, overseeing an array of stakeholders including representatives from technology, donor and academic institutions. Beyond this entity, a national oversight and enforcement regulatory body, the POPI Regulator for South Africa has been created to accommodate direct-to-consumer engagement such as the lodging of complaints and adjudication where uncertainty exists around legality of data management or use.^11^ In box 2, we outline some of the implications of POPI implementation for MomConnect. To support special requests for data processing, additional layers of ethical review may be required.

Increasingly, Institutional Review Boards (IRBs) need to become conversant in ethical considerations for digital datasets—which in some cases can be generated from electronic mobile health records without participants’ knowledge. Particularly where other legislative standards are not in place or are not enforced, IRBs are the gatekeepers and the last line of defense for protection of participants. Because they play such an important role, it is essential that IRB members keep abreast of key issues arising in mobile health environments and that they remain current in their understanding of the evolving landscape in digital health.

## Conclusion

The rapid proliferation of digital health tools globally and throughout LMICs offers much promise in addressing critical gaps in health systems. While there is a clear responsibility for researchers, programme managers and staff to ensure good digital data governance and appropriate, consented digital data use, there is also a very important role for governments and multinational bodies to define and demand appropriate digital data governance checks and balances in ongoing programmes. Here, we present a governance framework for digital health data in health systems research, that presents tenets of data governance at the micro level where interactions between individuals enable participant protection through consent processes and ethical engagement with personal data; at the meso level whereby organisations such as ethics review boards, donors and data access committees work together to ensure appropriate data use; and at the macrolevel whereby legislators and governments define how data governance must be undertaken to ensure the protection of individuals.

While we have sought to provide a starting point for this discussion, the global community would benefit from WHO guidance in this area. In the meantime, country level efforts are underway to push for greater accountability and transparency in the data governance structures and ethics procedures underpinning digital tools being implemented in the health sector. In India, the National Health Systems Resource Centre (NHSRC) has established a Community of Research and Practice for Digital Health which is developing impartial standards for assessing digital tools for frontline health workers, including data governance and ethics. Through a south to south collaboration of key stakeholders, we hope to extend these discussions to South Africa and stakeholders throughout the region with the broader aim of catalysing evidence based decision making as a part of strengthening the governance of scaling up of digital tools in the health sector.
