# Supplementary material for: How to use relevant data for maximal benefit with minimal risk: digital health data governance to protect vulnerable populations in low-income and middle-income countries
Source: BMJ Glob Health. 2019 Apr 11;4(2):e001395. doi: 10.1136/bmjgh-2019-001395 (PMC6509603; doi:10.1136/bmjgh-2019-001395)
Supplement: Supplementary data [file bmjgh-2019-001395supp001.pdf]

Supplementary table S1. Examples of content areas addressed by legislation across countries

Content areas		*EU	*SA
Participant protection	Requirements for informed consent are explicitly outlined	x	x
	Acceptable data uses are described to participants	x	x
	Risk to the participants are detailed	x	x
	Individuals can refuse or withdraw consent without detriment	x	x
	Individual "rights to be forgotten" are specified	x	x
	Procedures for directly verifying consent from individual are specified	x	x
Protection for vulnerable populations	Special categories of persons which merit higher protection are specified	x	x
Data access controls	Individual control over their own data including data elements collected, usage of those data, storage and deletion, are described	x	x
	Provisions are specified for accessing collected data, obtaining information on its use, and controlling access	x	x
	Provisions for correcting inaccuracies in data collected are outlined	x	x
	Parameters on the geographic location and movement of data are described	x	x
	Responsibilities for data security are detailed	x	x
	Provisions for data use exceptions are specified	x	x
Data use for research	Explicit guidelines for the use of data for research are described	x	x
Interoperability	Recommendations on data portability and interoperability are provided	x	
Accountability	The responsible parties for overseeing data management (collection, access, use) are explicitly defined	x	x
	Procedures for personal data breach are outlined	x	x
	Protocol for notifying participants of data breach is specified	x	x
	Categories of sensitive data types of data, including health, are defined	x	x
Unique circumstances	Requirements for data of deceased individuals are specified	x	x
	Requirements for data of vulnerable populations are specified (pediatric, elderly, HIV, mentally impaired, other conditions)	x	x

** EU: European Union. SA: South Africa*

Supplementary Table S2. Comparison and explanation of key terms used in GDPR and POPI

**GDPR** [34]	**POPI** [27]	**Interpretation**
Biometric Data	Biometrics	A technique of personal identification that is based on physical, physiological or behavioural characterisation including blood typing, fingerprinting, DNA analysis, retinal scanning and voice recognition.
Consent	Consent	Voluntary, specific and informed expression of will in terms of which permission is given for the processing of personal information.
Data Controller	Responsible Party	Entity or person(s) who determine, alone or with others, the purposes, conditions and means for processing personal information.
Data Erasure<br>Right To Be Forgotten	-	The right of the data subject to have the data controller erase his/her personal data, cease further dissemination of the data, and potentially have third parties cease processing of the data
-	Correction of Personal Information	A data subject to request correction or deletion of inaccurate, irrelevant, excessive, out of date, incomplete, misleading or unlawfully obtained personal information; or destruction/deletion of personal information that the responsible party is no longer authorised to retain
Right to Access<br>Subject Access Right	Access to Personal Information	The right of the data subject to know personal information concerning them held by a responsible party.
Data Portability	-	The right of the data subject to obtain and re-use data that is held on them, which must be received “in a structured, commonly used and machine-readable format”
Data Processor	Operator	An entity who processes personal information on behalf of a Responsible Party or Data Controller, rather than as a direct member or under the direct authority of that party
Data Protection Authority	Information Regulator	National authority who will ensure protection of the right to data protection, through monitoring and enforcement of the data protection act.
Data Protection Officer	Information Officer	An expert in data privacy who works with an independent entity to ensure their adherence to the act.
Supervisory Authority	-	Independent public authorities in EU Member States responsible for monitoring application of regulation.
Data Subject	Data Subject	Person to whom personal information relates
Anonymised data	De-identified data	Personal data with all components removed that identify, or could conceivable be used to re-identify a person directly or through linkage to other data
Pseudonymised Data	-	Data that can be re-identified and linked to a person either directly or through linkage to other data
Recipient	Recipient	The person(s) or entity receiving personal information.
Right to Access<br>Subject Access Right	Right of Access	The right of any individual to be able to access personal information held by a Controller or Responsible Party, pertaining to themselves.

Supplementary Table S3. Comparison of EU and South African legislation language on consent and data processing

	**European Union**	**South Africa**
Informed consent	(32) Consent should be given by a clear affirmative act establishing a freely given, specific, informed and unambiguous indication of the data subject's agreement to the processing of personal data relating to him or her, such as by a written statement, including by electronic means, or an oral statement. This could include ticking a box when visiting an internet website, choosing technical settings for information society services or another statement or conduct which clearly indicates in this context the data subject's acceptance of the proposed processing of his or her personal data. Silence, pre-ticked boxes or inactivity should not therefore constitute consent. Consent should cover all processing activities carried out for the same purpose or purposes. When the processing has multiple purposes, consent should be given for all of them. If the data subject's consent is to be given following a request by electronic means, the request must be clear, concise and not unnecessarily disruptive to the use of the service for which it is provided.<br>(43) In order to ensure that consent is freely given, consent should not provide a valid legal ground for the processing of personal data in a specific case where there is a clear imbalance between the data subject and the controller, in particular where the controller is a public authority and it is therefore unlikely that consent was freely given in all the circumstances of that specific situation. Consent is presumed not to be freely given if it does not allow separate consent to be given to different personal data processing operations despite it being appropriate in the individual case, or if the performance of a contract, including the provision of a service, is dependent on the consent despite such consent not being necessary for such performance.<br><br><u>Article 7: Conditions for consent</u><br>1. Where processing is based on consent, the controller shall be able to demonstrate that the data subject has consented to processing of his or her personal data.<br>2. If the data subject's consent is given in the context of a written declaration which also concerns other matters, the request for consent shall be presented in a manner which is clearly distinguishable from the other matters, in an intelligible and easily accessible form, using clear and plain language. Any part of such a	<u>Condition 2: Processing Limitation</u><br>Consent, justification and objection<br>11. (2) *(a)* The responsible party bears the burden of proof for the data subject's or *(b)* The data subject or competent person may withdraw his, her or its consent, as referred to in subsection (1)*(a)*, at any time: Provided that the lawfulness of the processing of personal information before such withdrawal or the processing of personal information in terms of subsection (1)*(b)* to *(f)* will not be affected.<br>13. (1) Personal information must be collected for a specific, explicitly defined and lawful purpose related to a function or activity of the responsible party.<br>(2) Steps must be taken in accordance with section 18(1) to ensure that the data subject is aware of the purpose of the collection of the information unless the provisions of section 18(4) are applicable.

	declaration which constitutes an infringement of this Regulation shall not be binding.<br>3. The data subject shall have the right to withdraw his or her consent at any time. The withdrawal of consent shall not affect the lawfulness of processing based on consent before its withdrawal. Prior to giving consent, the data subject shall be informed thereof. It shall be as easy to withdraw as to give consent.<br>4. When assessing whether consent is freely given, utmost account shall be taken of whether, *inter alia*, the performance of a contract, including the provision of a service, is conditional on consent to the processing of personal data that is not necessary for the performance of that contract.	
Data uses are described to participants	(39) Any processing of personal data should be lawful and fair. It should be transparent to natural persons that personal data concerning them are collected, used, consulted or otherwise processed and to what extent the personal data are or will be processed. The principle of transparency requires that any information and communication relating to the processing of those personal data be easily accessible and easy to understand, and that clear and plain language be used. That principle concerns, in particular, information to the data subjects on the identity of the controller and the purposes of the processing and further information to ensure fair and transparent processing in respect of the natural persons concerned and their right to obtain confirmation and communication of personal data concerning them which are being processed. …. Personal data should be processed in a manner that ensures appropriate security and confidentiality of the personal data, including for preventing unauthorised access to or use of personal data and the equipment used for the processing.<br><br><u>Section 2. Article 13</u><br>1. Where personal data relating to a data subject are collected from the data subject, the controller shall, at the time when personal data are obtained, provide the data subject with all of the following information:<br>(a) the identity and the contact details of the controller and, where applicable, of the controller's representative;<br>(b) the contact details of the data protection officer, where applicable;<br>(c) the purposes of the processing for which the personal data are intended as well as the legal basis for the processing;<br>(d) where the processing is based on point (f) of Article 6(1), the legitimate interests pursued by the controller or by a third party;	<u>Condition 6: Openness</u><br>Notification to data subject when collecting personal information<br>18. (1) If personal information is collected, the responsible party must take reasonably practicable steps to ensure that the data subject is aware of—<br>*(a)* the information being collected and where the information is not collected from the data subject, the source from which it is collected;<br>*(b)* the name and address of the responsible party;<br>*(c)* the purpose for which the information is being collected;<br>*(d)* whether or not the supply of the information by that data subject is voluntary or mandatory.

	(e) the recipients or categories of recipients of the personal data, if any;<br>(f) where applicable, the fact that the controller intends to transfer personal data to a third country or international organisation and the existence or absence of an adequacy decision by the Commission, or in the case of transfers referred to in Article 46 or 47, or the second subparagraph of Article 49(1), reference to the appropriate or suitable safeguards and the means by which to obtain a copy of them or where they have been made available.	
Risk to the participants	(39) …. Natural persons should be made aware of risks, rules, safeguards and rights in relation to the processing of personal data and how to exercise their rights in relation to such processing. In particular, the specific purposes for which personal data are processed should be explicit and legitimate and determined at the time of the collection of the personal data….<br>(76) The likelihood and severity of the risk to the rights and freedoms of the data subject should be determined by reference to the nature, scope, context and purposes of the processing. Risk should be evaluated on the basis of an objective assessment, by which it is established whether data processing operations involve a risk or a high risk.	Not specified
Refusal and/ or withdraw consent	(42) …. Consent should not be regarded as freely given if the data subject has no genuine or free choice or is unable to refuse or withdraw consent without detriment.	The data subject or competent person may withdraw his, her or its consent, as referred to in subsection (1)*(a)*, at any time: Provided that the lawfulness of the processing of personal information before such withdrawal or the processing of personal information in terms of subsection (1)*(b)* to *(f)* will not be affected.
Right to erasure ('right to be forgotten')	Article 17.<br>1. The data subject shall have the right to obtain from the controller the erasure of personal data concerning him or her without undue delay and the controller shall have the obligation to erase personal data without undue delay where one of the following grounds applies:<br>(a) the personal data are no longer necessary in relation to the purposes for which they were collected or otherwise processed;<br>(b) the data subject withdraws consent on which the processing is based according to point (a) of Article 6(1), or point (a) of Article 9(2), and where there is no other legal ground for the processing;<br>(c) the data subject objects to the processing pursuant to Article 21(1) and there are no overriding legitimate grounds for the processing, or the data subject objects to the processing pursuant to Article 21(2);<br>(d) the personal data have been unlawfully processed;	Not specified

	(e) the personal data have to be erased for compliance with a legal obligation in Union or Member State law to which the controller is subject;<br>(f) the personal data have been collected in relation to the offer of information society services referred to in Article 8(1).<br><br>2. Where the controller has made the personal data public and is obliged pursuant to paragraph 1 to erase the personal data, the controller, taking account of available technology and the cost of implementation, shall take reasonable steps, including technical measures, to inform controllers which are processing the personal data that the data subject has requested the erasure by such controllers of any links to, or copy or replication of, those personal data.	
Individual rights to correct inaccuracies/ ensure data quality	(59) Modalities should be provided for facilitating the exercise of the data subject's rights under this Regulation, including mechanisms to request and, if applicable, obtain, free of charge, in particular, access to and rectification or erasure of personal data and the exercise of the right to object. The controller should also provide means for requests to be made electronically, especially where personal data are processed by electronic means. The controller should be obliged to respond to requests from the data subject without undue delay and at the latest within one month and to give reasons where the controller does not intend to comply with any such requests.	<u>Condition 5: Quality of information</u><br>A responsible party must take reasonably practicable steps to ensure that the personal information is complete, accurate, not misleading and updated where necessary.<br>Correction of personal information<br>24. (1) A data subject may, in the prescribed manner, request a responsible party to— *(a)* correct or delete personal information about the data subject in its possession or under its control that is inaccurate, irrelevant, excessive, out of date, incomplete, misleading or obtained unlawfully; or *(b)* destroy or delete a record of personal information about the data subject that the responsible party is no longer authorised to retain.
Procedures for directly verifying consent	(42) Where processing is based on the data subject's consent, the controller should be able to demonstrate that the data subject has given consent to the processing operation. In particular in the context of a written declaration on another matter, safeguards should ensure that the data subject is aware of the fact that and the extent to which consent is given…..For consent to be informed, the data subject should be aware at least of the identity of the controller and the purposes of the processing for which the personal data are intended….	The responsible party bears the burden of proof for the data subject's or (2) *(a)* competent person's consent as referred to in subsection (1)*(a)*.
Processing of information	(33) It is often not possible to fully identify the purpose of personal data processing for scientific research purposes at the time of data collection. Therefore, data subjects should be allowed to give their consent to certain areas of scientific research when in keeping with recognised ethical standards for scientific research. Data subjects should have the opportunity to give their consent only to certain areas of research or parts of research projects to the extent allowed by the intended purpose.	Chapter 3: Conditions for lawful processing of personal information; Excerpts from Section 11<br><u>Condition 2. Consent, justification, and objection.</u><br>(3) A data subject may object, at any time, to the processing of personal information—<br>*(a)* in terms of subsection (1)*(d)* to *(f)*, in the prescribed manner, on reasonable grounds relating to his, her or its

	(40) In order for processing to be lawful, personal data should be processed on the basis of the consent of the data subject concerned or some other legitimate basis, laid down by law, either in this Regulation or in other Union or Member State law as referred to in this Regulation, including the necessity for compliance with the legal obligation to which the controller is subject or the necessity for the performance of a contract to which the data subject is party or in order to take steps at the request of the data subject prior to entering into a contract.<br>(60) The principles of fair and transparent processing require that the data subject be informed of the existence of the processing operation and its purposes. The controller should provide the data subject with any further information necessary to ensure fair and transparent processing taking into account the specific circumstances and context in which the personal data are processed. Furthermore, the data subject should be informed of the existence of profiling and the consequences of such profiling. Where the personal data are collected from the data subject, the data subject should also be informed whether he or she is obliged to provide the personal data and of the consequences, where he or she does not provide such data. That information may be provided in combination with standardised icons in order to give in an easily visible, intelligible and clearly legible manner, a meaningful overview of the intended processing. Where the icons are presented electronically, they should be machine-readable.<br><br>(70) Where personal data are processed for the purposes of direct marketing, the data subject should have the right to object to such processing, including profiling to the extent that it is related to such direct marketing, whether with regard to initial or further processing, at any time and free of charge. That right should be explicitly brought to the attention of the data subject and presented clearly and separately from any other information.<br><br><u>Section 2. Article 13</u><br>…..The controller shall, at the time when personal data are obtained, provide the data subject with the following further information necessary to ensure fair and transparent processing:<br>(a) the period for which the personal data will be stored, or if that is not possible, the criteria used to determine that period;<br>(b) the existence of the right to request from the controller access to and rectification or erasure of personal data or restriction of processing concerning the data subject or to object to processing as well as the right to data portability;	particular situation, unless legislation provides for such processing; or<br>*(b)* for purposes of direct marketing other than direct marketing by means of unsolicited electronic communications as referred to in section 69.<br>(4) If a data subject has objected to the processing of personal information in terms of subsection (3), the responsible party may no longer process the personal information.<br><u>Condition 3: Purpose specification</u><br>Personal information may only be processed if the data subject or a competent person where the data subject is a child consents to the processing;<br>processing is necessary to carry out actions for the conclusion or performance of a contract to which the data subject is party;<br>processing complies with an obligation imposed by law on the responsible party;<br>processing protects a legitimate interest of the data subject;<br>processing is necessary for the proper performance of a public law duty by a public body; or processing is necessary for pursuing the legitimate interests of the responsible party or of a third party to whom the information is supplied.<br><u>Condition 7: Security safeguards</u><br>Information processed by operator or person acting under authority<br>20. An operator or anyone processing personal information on behalf of a responsible party or an operator, must—<br>*(a)* process such information only with the knowledge or authorisation of the responsible party; and<br>*(b)* treat personal information which comes to their knowledge as confidential and must not disclose it, unless required by law or in the course of the proper performance of their duties.

	(c) where the processing is based on point (a) of Article 6(1) or point (a) of Article 9(2), the existence of the right to withdraw consent at any time, without affecting the lawfulness of processing based on consent before its withdrawal;<br>(d) the right to lodge a complaint with a supervisory authority;<br>(e) whether the provision of personal data is a statutory or contractual requirement, or a requirement necessary to enter into a contract, as well as whether the data subject is obliged to provide the personal data and of the possible consequences of failure to provide such data;<br>(f) the existence of automated decision-making, including profiling, referred to in Article 22(1) and (4) and, at least in those cases, meaningful information about the logic involved, as well as the significance and the envisaged consequences of such processing for the data subject.<br>3. Where the controller intends to further process the personal data for a purpose other than that for which the personal data were collected, the controller shall provide the data subject prior to that further processing with information on that other purpose and with any relevant further information as referred to in paragraph 2.	
Access to personal information	<u>Article 15. Right of access by the data subject</u><br>1. The data subject shall have the right to obtain from the controller confirmation as to whether or not personal data concerning him or her are being processed, and, where that is the case, access to the personal data and the following information:<br>(a) the purposes of the processing;<br>(b) the categories of personal data concerned;<br>(c) the recipients or categories of recipient to whom the personal data have been or will be disclosed, in particular recipients in third countries or international organisations;<br>(d) where possible, the envisaged period for which the personal data will be stored, or, if not possible, the criteria used to determine that period;<br>(e) the existence of the right to request from the controller rectification or erasure of personal data or restriction of processing of personal data concerning the data subject or to object to such processing;<br>(f) the right to lodge a complaint with a supervisory authority;<br>(g) where the personal data are not collected from the data subject, any available information as to their source;<br>(h) the existence of automated decision-making, including profiling, referred to in Article 22(1) and (4) and, at least in those cases, meaningful information about	23. (1) A data subject, having provided adequate proof of identity, has the right to— *(a)* request a responsible party to confirm, free of charge, whether or not the responsible party holds personal information about the data subject; and<br>*(b)* request from a responsible party the record or a description of the personal information about the data subject held by the responsible party, including information about the identity of all third parties, or categories of third parties, who have, or have had, access to the information—<br>(i) within a reasonable time;<br>(ii) at a prescribed fee, if any;<br>(iii) in a reasonable manner and format; and<br>(iv) in a form that is generally understandable.

	the logic involved, as well as the significance and the envisaged consequences of such processing for the data subject.<br>2. Where personal data are transferred to a third country or to an international organisation, the data subject shall have the right to be informed of the appropriate safeguards pursuant to Article 46 relating to the transfer.<br>3. The controller shall provide a copy of the personal data undergoing processing. For any further copies requested by the data subject, the controller may charge a reasonable fee based on administrative costs. Where the data subject makes the request by electronic means, and unless otherwise requested by the data subject, the information shall be provided in a commonly used electronic form.<br>4. The right to obtain a copy referred to in paragraph 3 shall not adversely affect the rights and freedoms of others.	
